# A randomized control trial to support smoke-free policy compliance in public housing

**DOI:** 10.1186/s13063-023-07339-4

**Published:** 2023-08-22

**Authors:** Diana Hernandez, Farzana Khan, David Albert, Daniel Giovenco, Charles Branas, Linda Valeri, Ana Navas-Acien

**Affiliations:** 1 Sociomedical Sciences, Columbia Mailman School of Public Health, New York, USA; 2 Health Policy and Management, Columbia Mailman School of Public Health, New York, USA; 3 Epidemiology, Columbia Mailman School of Public Health, New York, USA; 4 Biostatistics, Columbia Mailman School of Public Health, New York, USA; 5 Environmental Health Sciences, Columbia Mailman School of Public Health, New York, USA

**Keywords:** Smoking, Secondhand smoke, Tobacco, Smoke-free policy, Public housing

## Abstract

**Background:**

Smoke-free housing policies in multiunit housing are increasingly widespread interventions to reduce smoking and secondhand smoke exposure. Little research has identified factors that impede compliance with smoke-free housing policies in low-income multiunit housing and test corresponding solutions.

**Methods:**

We are using an experimental design to test two compliance support interventions: (A) a “compliance through reduction (via relocation and reduction in personal smoking) and cessation” intervention targets households with smokers and involves support to shift smoking practices to areas beyond the apartment or building setting, reduce personal smoking, and deliver in-residence smoking cessation support services via trained peer educators and (B) a “compliance through resident endorsement” intervention involving voluntary adoption of smoke-free living environments through personal pledges, visible door markers, and/or via social media. We will compare randomly sampled participants in buildings that receive A or B or A plus B to the NYCHA standard approach.

**Discussion:**

This RCT addresses key gaps in knowledge and capitalizes on key scientific opportunities by (1) leveraging the federal mandate to ban smoking in a public housing system of more than sufficient size to conduct an adequately powered RCT; (2) expanding our understanding of smoke-free policy compliance beyond policy implementation by testing two novel treatments: (a) in-residence smoking cessation and (b) resident endorsement, while (3) addressing population and location-specific tobacco-related disparities. At the conclusion of the study, this RCT will have leveraged a monumental policy shift affecting nearly half a million NYC public housing residents, many of whom disproportionately experience chronic illness and are more likely to smoke and be exposed to secondhand smoke than other city residents. This first-ever RCT will test the effects of much-needed compliance strategies on resident smoking behavior and secondhand smoke exposure in multiunit housing.

**Trial registration:**

Clinical Trials Registered, NCT05016505. Registered on August 23, 2021.

## Administrative information

Note: the numbers in curly brackets in this protocol refer to SPIRIT checklist item numbers. The order of the items has been modified to group similar items (see http://www.equator-network.org/reporting-guidelines/spirit-2013-statement-defining-standard-protocol-items-for-clinical-trials/).

**Table:** 

Title {1}	A randomized controlled trial to support smoke-free policy compliance in public housing.
Trial registration {2a and 2b}.	ClinicalTrials.gov NCT05016505
Protocol version {3}	September 2022
Funding {4}	National Institutes of Health, National Cancer Institute
Author details {5a}	Diana Hernández, PhD Associate Professor Sociomedical Sciences, Columbia Mailman School of Public Health Farzana Khan, MPH Project Coordinator Sociomedical Sciences, Columbia Mailman School of Public Health David Albert, MPH, DDS Associate Professor Health Policy and Management Columbia Mailman School of Public Health Daniel Giovenco, PhD Assistant Professor Sociomedical Sciences Columbia Mailman School of Public Health Charles Branas, PhD Gelman Endowed Professor Epidemiology, Columbia Mailman School of Public Health Linda Valeri, PhD, Assistant Professor Biostatistics, Columbia Mailman School of Public Health Ana Navas-Acien, MD, PhD Professor Environmental Health Sciences Columbia Mailman School of Public Health
Name and contact information for the trial sponsor {5b}	Stephanie Land, PhD Email: stephanie.land@nih.gov National Institutes of Health
Role of sponsor {5c}	For this study, all decisions regarding the design of the study; the collection, management, analysis, and interpretation of data; writing of results; and submissions for publication will be at the discretion of the principal investigator and research team. The study sponsor, NCI/NIH, will not contribute to, nor have authority, over any of these activities.

## Introduction

### Background and rationale {6a}

Tobacco use remains a public health priority, especially for racial/ethnic minorities and low-income populations [1]. Evidence demonstrating the adverse health effects of smoking has led to comprehensive tobacco control policies including smoking bans in public venues [2]. Such bans provide protection from secondhand smoke, reduce smoking overall, and increase adoption of voluntary smoke-free rules in private homes [3–6], producing positive health effects, most notably in reducing asthma [7]. Prior to the COVID-19 pandemic, Americans spent, on average, 69% of their time at home [8], which has increased with the emphasis on staying home. Considering this, housing represents a major potential source of secondhand smoke exposure, and is, in fact, the largest source of secondhand smoke exposure for children [9]. Approaches to limit the spread of secondhand smoke in indoor spaces—such as separation of smokers and non-smokers, ventilation, or cleaning the air—cannot fully eliminate exposure [10, 11]. Building-wide bans on indoor smoking go further to achieve this goal.

Individuals’ private homes have traditionally been considered outside the scope of appropriate smoking regulations [12]. However, the case for smoke-free housing has been made convincingly enough [13–15] to prompt the United States Department of Housing and Urban Development (HUD) to mandate that all public housing developments adopt such policies [16]. These policies carry potential economic, environmental, and health benefits to residents and owners [17], particularly in multiunit housing.

It should be noted that disadvantaged groups are more likely to live in multiunit housing. Residents of multiunit dwellings have reported higher smoking [18] and exposure to secondhand tobacco smoke largely due to air exchange between apartments through mechanisms such as ventilation systems, common areas, and contiguous party walls [17, 19]. In fact, an estimated 28 million multiunit housing residents with smoke-free rules in their unit experience secondhand smoke exposure each year [20], and secondhand smoke incursions into residential units are noted whether measured by self-reports, environmental markers, or biomarkers [21–23].

Moreover, in a key study, 89% of non-smoking households were exposed to secondhand smoke resulting in involuntary smoking activity as high as one cigarette per day [24–28]. Non-smoking multiunit housing residents, both adults and children, exhibited significantly elevated levels of cotinine, a biomarker of cigarette smoke exposure [21, 24–28], compared to non-smokers living in detached homes [26, 29]. Similarly, 84.5% of children living in apartments without any household members who smoked inside “had a cotinine level that indicated recent tobacco smoke exposure.” One cause for this finding is the seepage through ventilation systems or walls from neighboring apartments where smoking took place [19, 30–35]. These studies all conclude that smoke-free housing policies involving building-wide bans on indoor smoking would effectively reduce secondhand smoke exposure [19, 21, 23–35].

Early studies of smoke-free housing policies have shown some protections from smoking hazards for smokers and non-smokers alike through decreased indoor secondhand smoke exposure, decreased smoking overall, and increased quit attempts [22, 24–28]. Yet, compliance with smoke-free housing policies is not assured [21]. For example, one study found that about half of residents or their guests had violated the policy [29]. In one study, almost 20% of residents reported “frequent exposure” to secondhand smoke after smoke-free housing policy implementation [26], and non-smoker residents still frequently experience secondhand smoke incursions [19, 30–35]. Another study found that though there were reductions in environmental markers of secondhand smoke initially, there was a 33% increase post 12 months of the policy being implemented [36] while another study found no change 12 months post implementation [37].

Hernández (PI) explored the multifaceted reasons for non-compliance, captured in the “social contract of smoke-free housing” concept which describes factors such as social ties and bi-directional rights and obligations between residents and property management that play a role in compliance [38]. A study assessing resident opinions on the smoke-free policy prior to implementation found 65% of residents supported the policy [39]. However, residents also expressed expectations of poor policy compliance due to lack of enforcement, safety concerns/inconvenience with smoking relocation, and general discontent with their housing authority and/or living conditions [39–41]. Such concerns also remain consistent post policy implementation [42, 43]. A mixed methods study focusing on resident experiences pre and post policy implementation found residents still reported smoking violations 1 year after the policy was implemented. Participants in the study reported that the policy overreached by telling people what to do, was inconsistently enforced, and created the perception that smokers were being unfairly targeted given that other pressing housing issues were not addressed [42]. Support for the policy remained unchanged for the study highlighting residents’ willingness to accept the policy, but the finding of a decline in satisfaction with enforcement indicated a need to address contextual barriers to compliance [42]. Continued exposure to secondhand smoke further poses a threat to overall satisfaction of housing with those with continued secondhand smoke exposure reporting lower satisfaction of housing conditions when repairs and improvements are made [44]. Further, it was found that compliance is often realized in an effort to avoid punishment rather than a genuine wish to comply and that receiving support as opposed to penalization is essential [40]. A Detroit-based study found that though some public housing residents had a desire to quit, limited cessation services, lack of access to medications, and social triggers made it difficult [45]. Another study found that 48.6% of 233 cigarette smokers thought about quitting specifically in response to the policy 12 months post implementation [46]. Overall, these studies demonstrate a general consensus that providing smoking cessation services would be beneficial in supporting compliance to the smoke-free mandate. Further evidence suggests that consistent engagement of residents in the process of implementation could support increased compliance [36, 42–44, 47]. Though these strategies have been suggested, no study to date provides a comprehensive understanding and testing of effective compliance support strategies for smoke-free policies.

This randomized control trial (RCT) addresses key gaps in knowledge and capitalizes on unique scientific opportunities that (1) leverage the HUD smoking ban within a large public housing system that will enable an adequately powered RCT; (2) expand understanding beyond smoke-free policy implementation to focus on compliance and effectiveness, and (3) address population and location-specific tobacco disparities.

### Objectives {7}

The broad objective of this study is to use an experimental design to test two smoke-free policy compliance support interventions: (A) a “compliance through reduction (via relocation and reduction in personal smoking) and cessation” intervention targets households with smokers and involves support to shift smoking practices outside of apartment and building settings and reducing personal smoking, and deliver in-residence smoking cessation support services via trained peer educators and (B) a “compliance through resident endorsement” intervention involving voluntary adoption of smoke-free living environments through personal pledges, visible door markers, and/or via social media. We will compare randomly sampled participants in buildings that receive A or B or A plus B to the NYCHA standard approach.

We seek to achieve the following specific aims:

- Aim 1: Experimentally test if compliance support interventions reduce personal smoking behavior.
- Aim 2: Experimentally test if compliance support interventions reduce secondhand smoke exposure.
- Aim 3: Determine if the tobacco retail environment surrounding each building moderates the relationship between compliance support interventions and smoking-related outcomes.

We hypothesize that the reduction/cessation plus resident endorsement intervention will yield significantly larger reductions in personal smoking and secondhand smoke exposure, compared to standalone interventions and the standard approach.

### Trial design {8}

**Fig. 1:**
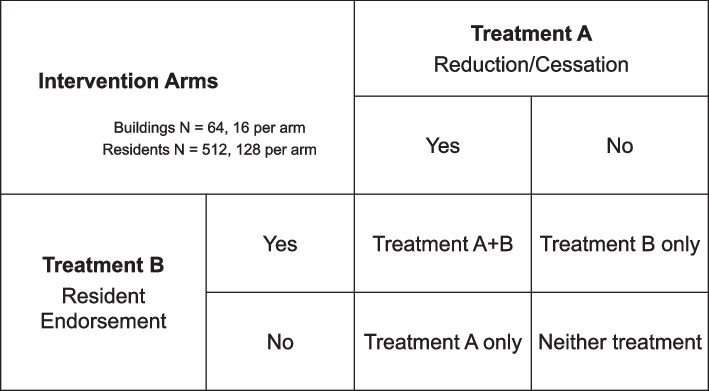
Intervention arms

The trial design is a borough-stratified, four-arm, factorial-design, cluster RCT that targets 64 randomly selected buildings (16 buildings per arm) in separate NYCHA developments. We are recruiting and following 8 randomly selected residents stratified by smoking status—4 smokers and 4 non-smokers—per building (*n*=512) into four arms: (1) reduction/cessation, (2) resident endorsement, (3) reduction/cessation plus resident endorsement, and (4) the standard approach (128 participants per arm) as shown in Fig. 1. A partnering community-based organization, Health People, Inc., specializing in peer-to-peer health education train and supervise peer educators (PEs) who deliver the designated interventions. Quantitative assessments via survey questionnaire, salivary cotinine measurements, and sensory building observations will be used to measure outcomes. We will be evaluating the efficacy of compliance support interventions in reducing personal smoking and exposure to secondhand smoke in public housing. In addition, we will also determine if the tobacco retail environment surrounding a given public housing building moderates the relationship between compliance support interventions and smoking-related outcomes.

**Fig. 2:**
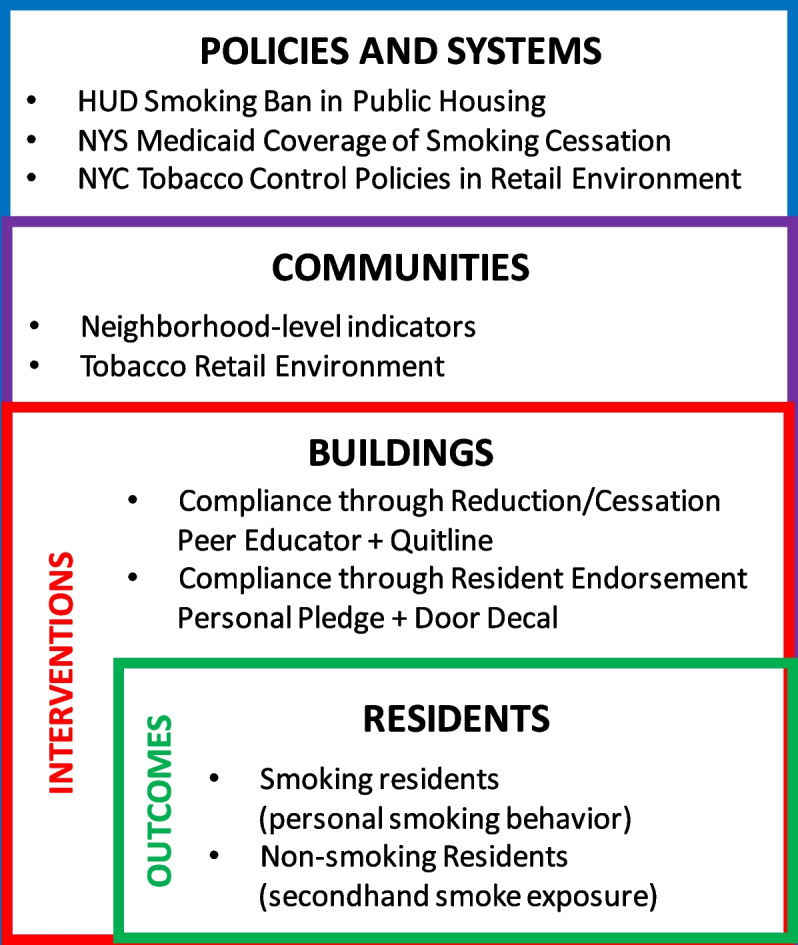
Conceptual framework

This study applies the *Social Ecological Theory, *which posits that multiple intersecting levels of influence affect human behavior and actions [48], to provide a more comprehensive understanding of smoking, cessation, and tobacco-related health disparities [49] including individual, relationship, community, and societal level of influences. Applying this theory to our study, public housing residents are influenced by interpersonal interactions among residents (micro-level), secondhand smoke exposures within residential settings (meso-level), and tobacco retail environment and tobacco control policies (macro-level). Previous work has targeted each of these aspects for smoking cessation interventions [47, 50–52], examined the impact of smoke-free air laws on quitting intentions [53], and found that the perception of social capital and social participation are associated with increased smoking cessation [54–56]. This resonates with the strategies in the resident endorsement intervention where social capital and social participation are foundational to the success of the intervention to gain tenant support for the smoke-free policy. However, our interventions are uniquely designed to extend beyond what has been found in prior work by capturing multiple facets of this theory by targeting various levels simultaneously (see Fig. 2) [51, 57, 58], reducing barriers to the use of counseling and medication in quit attempts [59], and leveraging social capital and social participation in housing communities to reduce individual smoking behaviors and exposures to secondhand smoke [60–63].

## Methods: participants, interventions and outcomes

### Study setting {9}

The New York City Housing Authority (NYCHA) is the largest public housing provider in the US with over 500,000 residents across 335 developments throughout the city via conventional public housing, Section 8, and Permanent Affordability Commitment Together (PACT) or Rental Assistance Demonstration (RAD) housing [64]. Though NYCHA is unique in its size and organization of buildings, it is characteristic of other public housing settings in that its entire portfolio of buildings and campuses are subject to smoking bans. Additionally, the resident profiles are similar across socioeconomic status and smoking behaviors. For feasibility, this study is being conducted at public housing sites located across two New York City boroughs where public housing is most concentrated: Manhattan and the Bronx. There are a total of 159 developments and 93,012 current dwelling apartments in these two boroughs. Of these, we have restricted the pool of buildings to contain only buildings managed by NYCHA. Further building inclusion/exclusion criteria are detailed below.

### Eligibility criteria {10}

There are two sets of eligibility criteria: (1) building eligibility and (2) participant eligibility.

The first stage of enrollment occurs at the building level as the buildings are randomized to receive a designated intervention (A, B, A+B) or act as a control site. Buildings must (1) have more than 50 units and (2) not be undergoing major renovations. Buildings that belong to developments that are (1) not in Manhattan or the Bronx, (2) smaller than 50 units, (3) undergoing major renovations, (4) mixed finance, (5) exclusively for elderly, (6) privately managed, (7) or will be part of Rental Assistance Demonstration (RAD) or Permanent Affordability Commitment Together (PACT) are excluded. RAD and PACT programs are associated with increased funds towards repairs and investments in social programming that have the potential to influence the impact of the designed interventions [65, 66], and, thus, have been excluded, see Hernández et al., for a study on resident smoking in the context of RAD  [44].

**Fig. 3:**
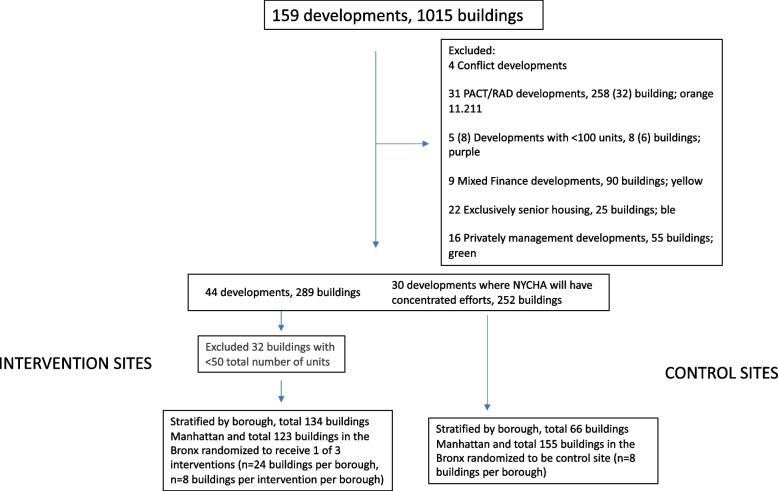
Development and building inclusion/exclusion process

Upon randomization, resident leaders are informed of the study on a rolling basis. If a resident leader declines participation of their buildings and residents in the study, their buildings are removed from the pool of eligible buildings. See Fig. 3 for the site selection process.

The second stage of enrollment is at the resident level. Participants must be (1) able and willing to provide verbal informed consent, (2) at least 18 years old, (3) able to communicate in English or Spanish, (4) living in the building at least 5 days/week and 9 months/year, and (5) not planning to move in the next 2 years. Participants who (1) have severe physical or mental medical conditions (e.g., cognitive disability) or other factors that could limit participation or ability to give informed consent in the study at baseline or during follow-up visits, (2) participate in focus groups conducted pre or concurrently as RCT that are geared towards understanding how to improve intervention arms for greater impact (buildings identified for focus groups are buildings that will not be assigned an intervention or have recruitment conducted for the main study), or (3) only smoke non-tobacco products (e.g., marijuana) are excluded from participating in the study due to the focus of the study on tobacco products. It should be noted that during the period of recruitment, the smoke-free policy in NYCHA was modified to also include marijuana use. In anticipation of this modification to the smoke-free policy, we are collecting preliminary information regarding marijuana use.

**Fig. 4:**
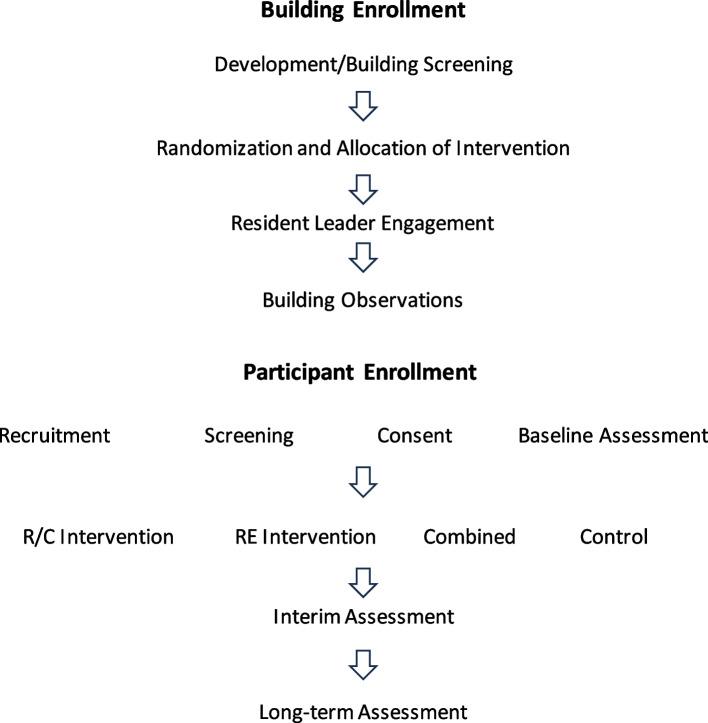
Building and participant enrollment process

Recruitment of NYCHA residents is conducted via door knocking and lobby intercepts until the targeted number per group is reached (4 smokers, 4 non-smokers in each building). Smoker status is defined as smoking a tobacco product including e-cigarettes at least 5×/month, and non-smoker status is defined as never having smoked any product or quit smoking at least 12 months prior to recruitment (Fig. 4).

### Who will take informed consent? {26a}

Participant contact is done face-to-face with systematic questionnaires. Potentially identifying information is asked or recorded for study participants (although NO birthdates, photos, or video recordings will be taken of participants) by trained field research support staff. Consent to participate in the study is asked before the actual study begins and individuals who refuse will not take part in the study. We explain the study, its intent and its potential risks, and ask if we can proceed. Informed consent documents and study materials is translated (forward and back translated) and administered in Latin Spanish where needed. Any information that is collected and is recorded first on paper is then entered into a computer and transferred and stored in a secure electronic environment. Upon conclusion of the study, all identifying information for each participant will be destroyed.

Additionally, a waiver of written documentation of informed consent has been obtained through the overseeing IRB as the research presents no more than minimal risk of harm to subjects and does not involve procedures for which written consent is normally required outside of the research context. A waiver of documentation of consent is appropriate because the only link between the subject and the study would be the consent document and the primary risk is a breach of confidentiality.

### Additional consent provisions for collection and use of participant data and biological specimens {26b}

The study data will be released to the funder, the National Institutes of Health (NIH) and National Cancer Institute (NCI) at the conclusion of the study. Identifiers will be removed and, after such removal, the information or biospecimens could be used for future research studies or distributed to another investigator for future research studies without additional informed consent, as appropriate.

## Interventions

### Explanation for the choice of comparators {6b}

Buildings and study participants assigned to the comparator arm [Arm 4] are recruited and followed over approximately a 12-month period to assess outcomes. No additional programs or services are delivered to the buildings or residents assigned to this arm beyond standard programs that NYCHA may provide to support the smoke-free mandate. Field staff document any policy-related signage, activities, or information to which these participants are exposed. We expect and have confirmed that Smoke-Free NYCHA’s liaison program will occur during our period of data collection during which they will conduct programming and activities that support compliance to the smoke-free policy. As such, the buildings enrolled in our “Standard NYCHA approach” are buildings that the Smoke-Free NYCHA team has identified as locations for concentrated efforts of their own smoke-free programming over the course of our data collection period. We will therefore evaluate the impact of this parallel prevention program including a covariate for NYCHA study participation at baseline in the models specified below. We will further allow for an interaction between the NYCHA study participation indicator and the treatment group.

### Intervention description {11a}

The development of the interventions is grounded on prior smoke-free policy endorsement and tobacco reduction/cessation programs. NYCHA conducted a smoke-free pilot project in 2015 centered on a smoke-free home pledge at a housing complex in Upper Manhattan [67]. The initiative was organized by members of the tenant association and entailed families and neighbors taking a voluntary pledge to maintain a smoke-free living environment. The overwhelming majority of residents (85%) signed the pledge, including nine floors in which all apartments took the pledge. At a culminating celebration, residents shared personal accounts of motivations for endorsing a smoke-free living environment [67]. Several elements of the “resident endorsement” intervention arm are fashioned after this pilot initiative, including fostering social connections between residents, providing information on the hazards of smoking and secondhand smoke along with the pledge culminating in a town hall session.

For the “reduction/cessation” intervention arm, Dr. David Albert, DDS, has led and directed the design of the tobacco cessation protocol. He also provided the foundational tools and trainings necessary to effectively use motivational interviewing to not only support smoking cessation, but also to reduce personal smoking. Dr. Albert’s expertise comes from implementing and evaluating evidence-based tobacco cessation programs at the New York Presbyterian (NYP) Cornell and NYP Columbia campuses and within affiliated community-based organizations, including many that engage minority populations. At NYP Hospital, he is the Tobacco Cessation Project Lead for the Medicaid Redesign Project funded by the NYDOH Delivery System Reform Incentive Payment Program. This program trains clinicians through a certified tobacco cessation specialist program, implements tobacco cessation counseling, and offers community presentations and engagement. As the Co-PI for the NCI funded Tobacco Cessation via Public Health Dental Clinics, he has examined the effectiveness of brief office-based interventions designed to assist patients quitting smoking or smokeless tobacco use. Dr. Albert is the co-Project Lead of the Development of a Tobacco Cessation Program for Cancer Patients at Columbia University. The project aims to implement an evidence-based tobacco cessation program for smoking patients at the Columbia University Herbert Irving Comprehensive Cancer Center.

To further enhance the intervention curriculum, we have conducted key informant interviews with residents and experts in resident engagement, tobacco cessation, and housing-based health interventions. These interviews were designed to elicit strategies to not only improve the RCT approach, but also to ascertain necessary adaptations to the intervention protocols to maximize their efficacy. Such discussions led to expanding the scope of the reduction/cessation intervention which previously was designed to focus on relocating smoking and cessation only. Furthermore, our community partner, Health People, has previously conducted health education activities in similar settings and their expertise in resident facing activities will further enhance resident engagement.

Prior to the implementation of the intervention, recruitment, and baseline data collection via survey questionnaire, passive drool sample collection and sensory building observations are conducted. Trained study staff obtain verbal consent upon the time of recruitment and data collection prior to enrollment in the study. Approximately a month after this completion, interventions are implemented by peer educators in accordance with respective building assignments.

#### Arm 1: Reduction (via relocation and reduction in personal smoking)/cessation

The reduction/cessation arm (Arm 1) “meets smokers where they are” using (a) a harm reduction approach that reinforces the use of designated smoking areas available and/or identifies ways residents can reduce their personal smoking more generally via motivational interviewing skills thereby encouraging compliance via relocation and personal smoking reduction; along with (b) in-residence cessation support to reduce barriers to participation in cessation services. Enrolled participants who are smokers are referred by the survey team to peer educators from Health People. The peer educator coordinates smoking cessation support, including serving as a liaison between participant and research team, providing information regarding the smoke-free policy and opportunities for relocation, and connecting participant to tobacco replacement therapy and/or physician support if deemed appropriate.

#### Arm 2: Resident endorsement

The resident endorsement model (Arm 2) seeks to engage residents by using an empowerment/network-based approach to shift the culture of health in buildings that encourages residents to collectively tackle indoor smoking and secondhand smoke exposure via physical demarcations and/or social media. Buildings assigned to this arm are targeted for a series of 2 in-residence programs that involve community forums and the creative arts to garner resident endorsements of smoke-free living environments. Premised on resident engagement, this arm seeks to impact social and physical dimensions of the residential environment to achieve compliance. The sessions (1) inform residents of risks associated with smoking and secondhand smoke; (2) identify reasons to have a smoke-free home, (3) ask residents to sign a pledge on paper and/or virtually; (4) display smoke-free signage on doors and/or social media pages with an original hashtag (#Smokefree[building address]); and (5) refer residents to the Smoke-free NYCHA website for information on the policy and existing cessation resources.

#### Arm 3: Combined intervention

There are buildings assigned to receive both models (Arm 3) for which in-residence programs based on the resident endorsement treatment and the smoking reduction/cessation treatment are provided. Both occur simultaneously with one geared towards all building residents (resident endorsement) and the other targeting smokers (smoking reduction/cessation) with the goal of reducing both personal smoking and secondhand smoke exposure.

The interventions and comparison group are outlined in Table 1.

**Table 1:** Overview of interventions

Intervention details			
**Reduction and Cessation arm**	**Resident endorsement arm**	**Combined arm**	**Standard NYCHA approach arm**
• Individual level	• Building level	• Individual and building level	• Building level
• Peer educators will provide smoking cessation/reduction support, providing information regarding the smoke-free policy and opportunities for relocating smoking, and connecting participant to access to tobacco replacement therapy and/or physician support if deemed appropriate.	• Buildings assigned will be targeted for a series of 2 in-residence programs	• Will provide in-residence programs based on the resident endorsement treatment and the smoking reduction/cessation treatment	• No additional programs or services will be delivered to the buildings or residents assigned to this arm beyond standard programs that NYCHA may provide to support the smoke-free mandate. Field staff will document any policy-related signage, activities, or information to which these participants are exposed.
	• Will: (1) inform residents of risks associated with smoking and secondhand smoke; (2) identify reasons to have a smoke-free home, (3) ask residents to sign a pledge on paper and/or virtually; (4) display smoke-free signage on doors and/or social media pages with an original hashtag (#Smokefree[building address]); and (5) refer residents to the Smoke-free NYCHA website for information on the policy and existing cessation resources		

### Criteria for discontinuing or modifying allocated interventions {11b}

The criteria for discontinuing or modifying allocated interventions in any given building is in response to any requests by overseeing building authority/management who voice concerns about administering interventions and decline further participation of their building, and subsequently residents, in the study.

The criteria for discontinuing targeted intervention approaches like the reduction/cessation and combined interventions is in response to participant request to discontinue or nonresponse.

### Strategies to improve adherence to interventions {11c}

Interventions are administered by peer educators who collect evaluations to assess quality of interactions and sessions. These are reviewed by researchers periodically to ensure participants are engaging with the activities in a meaningful way and appropriate protocols in delivering the interventions are followed. Research team members also periodically attend sessions related to resident endorsement activities and meetings with participants related to the reduction/cessation activities to further ensure appropriate protocols are taken.

### Relevant concomitant care permitted or prohibited during the trial {11d}

Participants are not restricted from pursuing additional/further tobacco reduction/cessation support services. We make every effort to document any services provided by NYCHA during regular briefings, and also in assessments.

### Provisions for post-trial care {30}

For the reduction/cessation intervention, individuals interested in quitting are provided a month supply of nicotine patches. There are limited risks and steps are taken to ensure those more likely to experience possible side effects are not provided patches. Peer educators report any instance of harm to the Columbia research team who then take action to remedy the situation as deemed appropriate and report the adverse event accordingly.

### Outcomes {12}

#### Primary outcomes

There are three primary outcomes being measured:

1. We will evaluate the change in number of cigarettes smoked per day using self-reported average number of cigarettes smoked per day among smokers measured at the time of baseline assessment, interim assessment (approximately 3 months post intervention), and long-term assessment (approximately 12 months post intervention).
2. We will evaluate change in salivary cotinine levels for 25% of the sample via passive drool collection among smokers and non-smokers collected at baseline assessment, interim assessment, and the long-term assessment.
3. We will evaluate change in secondhand smoke exposure via self-reported amount of exposure to secondhand smoke. This will be measured in all three assessments.

#### Secondary outcomes

There are 6 secondary outcomes being measured:

1. We will evaluate change in number of participants with successful quit attempts measured at baseline assessment, interim assessment, and long-term assessment for smokers.
2. We will evaluate change in number of quit attempts using the mean number of quitting attempts among smokers measured at baseline, interim, and long-term assessments.
3. We will evaluate change in number of participants with secondhand smoke observations who have either observed secondhand smoking or not measured at baseline, interim, and long-term assessments.
4. We will evaluate change in number of hours of secondhand smoke exposure via self-reported number of hours of observing someone smoke indoors measured at baseline, interim, and long-term assessments.
5. We will evaluate change in number of smokers via counted number of people observed smoking in common areas at building visits.
6. We will evaluate change in number of cigarette butts via counted number of cigarette butts observed in common areas at building visits.

**Fig. 5:**
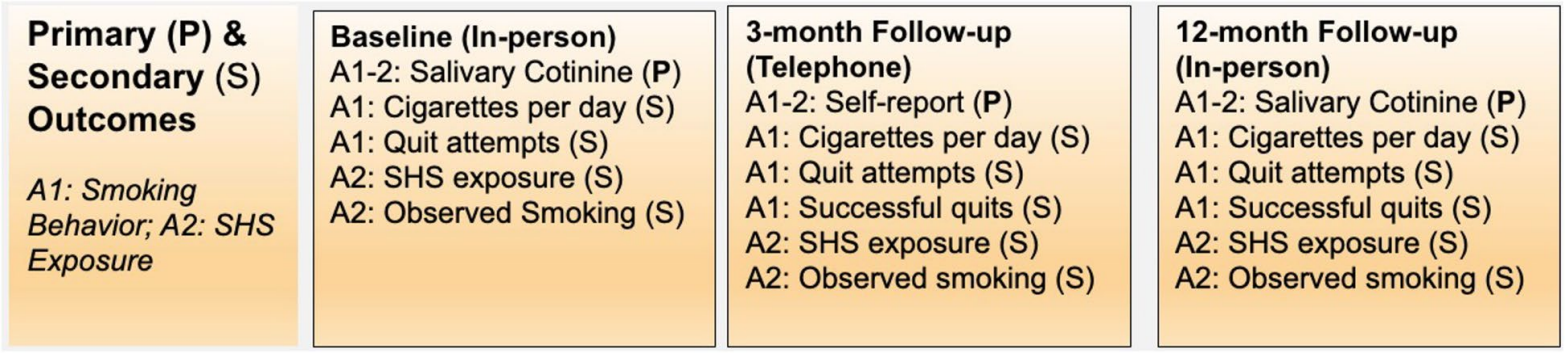
Primary and secondary outcomes

These outcome measures are summarized in Fig. 5.

### Participant timeline {13}

Consented, eligible participants are in the study for up to 15 months once recruited. A staggered recruitment, data collection, and intervention delivery model is implemented to ensure appropriate timing of participant involvement from recruitment to the final follow-up and to better align study resources across all 64 enrolled buildings (See Table 2.)

**Table 2:** Participant timeline

	**Participant timeline**				
	**Enrolment**	**Post-allocation**			**Close-out**
TIMEPOINT**	***−t***_***1***_	***t***_***0***_	***t***_***2***_	***t***_***5***_	***t***_***14***_
		***Baseline***		***3-month follow-up (post intervention)***	***12-month follow-up post intervention)***
**Building enrolment:**					
**Eligibility screen**	X				
**Resident leader engagement**	X				
**Building randomization**	X				
**Allocation to intervention**	X				
**Participant enrolment:**					
**Eligibility screen**		X			
**Informed consent**		X			
**Interventions:**					
***Reduction cessation***			X		
***Resident endorsement***			X		
**Assessments:**					
***Building observations***		X			X
***Saliva sample***		X			X
***Building observations***		X			X
***Survey***		X		X	X

### Sample size {14}

Power analysis was conducted using software PASS.15 for the primary outcome (change in salivary cotinine over a year) in Aims 1 and 2. Sampling 16 clusters (buildings) with 4 subjects per cluster (smokers for Aim 1, non-smokers for Aim 2) in each arm, we have 128 subjects per group or 256 in groups with or without a specific type of treatment (e.g., resident endorsement vs. no endorsement, cessation vs. no cessation). We calculated effect size as group mean difference in unit of standard deviation (SD) of the outcome, based on a two-sided test for mean difference between two groups in a cluster-randomized design with power of 80%. To compare groups with and without specific type of treatment, the group size of 256 may detect effect size of 0.283SD, 0.299SD, and 0.312SD at significance level alpha=0.05 for intra-cluster correlation coefficient *r*=0.1, 0.15, 0.2, respectively. To compare intervention arms to the control arm, we have 3 comparisons and use conservative Bonferroni adjustment for multiple tests on significance level that alpha= 0.0167 (=0.05/3). The sample size of 128 subject per arm may detect effect size of 0.472SD, 0.499SD, and 0.524SD for *r* = 0.1, 0.15, and 0.2, respectively, at alpha=0.0167. To put these numbers in perspective, using mean and SD estimated from a study of smokers in Maryland, an effect size of 0.283SD would correspond to a mean difference in salivary cotinine between the two intervention groups of 55.3 ng/mL with group SD= 195.4 ng/mL for smokers and of 0.19 ng/mL with group SD=0.672 ng/mL for non-smokers. For instance a mean difference in 5 cigarettes smoked per day in the same study was 59.6 ng/mL for smokers. In England, following the smoke-free legislation, salivary cotinine declined from mean level of 0.36 to 0.07 ng/mL (mean difference 0.29 ng/mL) among non-smokers. These findings support that we have sufficient power to estimate the impact of our interventions. The study is powered on an 80% retention rate, which we have achieved in other studies. Reasons for attrition will be tracked; those lost to follow-up will be compared to the final sample.

### Recruitment {15}

The primary method of recruitment is via door knocking and lobby intercepts until we reach our targeted number of residents per group (4 smokers, 4 non-smokers in each building). We make every attempt to retain participants and minimize attrition bias through a range of rapport building and retention techniques, incentives, and cultural adaptation of study materials. We have also powered the study based on 80% retention.

While the study presents minimal risks, participants may withdraw at any time upon request.

Alternatives available to participants outside the research context are simply not enrolling and attempting to serve the greater good by participating in other research or community service activities.

## Assignment of interventions: allocation

### Sequence generation {16a}

A list of eligible buildings from each borough was randomly ordered by computer-generated random numbers for each borough. Then, the first 24 buildings for each borough (*n*=48, 24 per borough) was randomly assigned to receive intervention A, B, or A+B.

Additionally, all buildings that are eligible for the study but have been identified by NYCHA as locations where they will conduct activities to promote the smoke-free policy were randomly ordered by computer-generated numbers for each borough. Then, the first 8 buildings for each borough (*n*=16, 8 per borough) was assigned to serve as a control site.

### Concealment mechanism {16b}

The allocation of interventions was done on a building basis and was only be revealed to specific research team members and intervention implementors (PEs). This information is passed to Health People via encrypted emails. All data collectors are not informed of intervention assignments for the duration of the study.

### Implementation {16c}

Dr. Valeri oversaw the allocation sequence and intervention assignment prior to the start of recruitment. Trained field-based research support staff are recruiting and enrolling participants in buildings that are enrolled in the study.

## Assignment of interventions: Blinding

### Who will be blinded {17a}

Trial participants and outcome assessors are blinded after the assignment to interventions. All interventions are assigned at the building level. We have a separate field team research support staff who conduct recruitment and collect the assessments but are unaware of the assignments.

### Procedure for unblinding if needed {17b}

Overall, the study has very minimal risks, and by its nature participants may know which intervention they/their building is receiving based on what services, if any, are provided and what materials are displayed, if any. Blinded data collectors may also guess the intervention based on what materials are displayed or not displayed. The intervention team, the peer educators will report any serious adverse events that occur during intervention implementation to the research team, but the identity of the participant and the intervention assigned to their building will not be revealed to data collectors.

## Data collection and management

### Plans for assessment and collection of outcomes {18a}

There are three assessments during this trial. These include a baseline assessment conducted upon recruitment and after building randomization, an interim assessment conducted over the phone approximately 3 months after the delivery of intervention, and a final long-term assessment conducted approximately 12 months after completion of the baseline assessment. The survey questionnaire collects information on housing conditions, indoor air quality, personal smoking behavior and cessation attempts, exposure to secondhand smoke, opinions on secondhand tobacco exposure, the smoke-free policy and tobacco policies, and health conditions. Additionally, passive drool samples from 25% of participants (*n*=128) is collected at the time of all three assessments. Sensory building observations occur at the start of recruitment and during the final assessments to record signs of active smoking and/or tobacco/marijuana odor. The presence of cigarettes, cigars, ashtrays with/out ashes, matches, or lighters will be documented within apartments and around the building premises including stairwells, elevators, hallways, laundry rooms, in/outdoor community spaces, playgrounds, and the perimeter of the buildings.

### Plans to promote participant retention and complete follow-up {18b}

We make every attempt to retain participants and minimize attrition bias through a range of rapport building and retention techniques, incentives, and cultural adaptation of study materials. Though primary outreach is conducted in-person, we also conduct mail and text outreach to maximize participant engagement. We have also powered the study based on 80% retention.

While the study presents minimal risks, participants may withdraw at any time upon request.

Alternatives available to participants outside the research context are simply not enrolling and attempting to serve the greater good by participating in other research or community service activities.

### Data management {19}

We use a digital data capture system and collect data on password-protected laptop computers. Specifically, this multi-user system is registered with CUIMC IT. Data is transferred, encrypted, backed up, and transferred to the data manager who review the forms daily for completeness.

Data is transferred via a firewall-protected, secure, electronic file-transfer to computer server space dedicated to the proposed study and protected with firewall and encryption technologies. Only Dr. Hernández, the Project PI, has master access to this server and she is required to authenticate herself via password each time the data is accessed.

Because this study is funded by the National Institutes of Health (NIH), the study is automatically issued a federal Certificate of Confidentiality. It is possible that participants will disclose information during their participation in the study and/or interviews and focus groups that indicates their involvement in illegal activities or indicates illegal activities by others. A Certificate of Confidentiality is one further means of ensuring that this information is kept confidential. Should participants disclose information in the middle of an interview that clearly indicates that they or those around them are in imminent danger, the interview will be suspended and 9-1-1 will be contacted.

Personally identifiable information (PII) is collected and entered into an electronic database for each study participant. This PII data is necessary to conduct the trial and include the following: name; street address, city, county, and zip code; telephone numbers; and possibly electronic mail addresses. All data is specifically used for the purposes of the proposed research. No PII is disclosed to anyone who is outside of the proposed research team and all PII will be destroyed when the study is concluded. We institute strict precautions and security procedures to maintain data integrity and confidentiality:

1. All data is stored on a centralized computer server at the Columbia University Mailman School of Public Health. This server is dedicated to the proposed study and is protected with up-to-date “fire wall” electronic security technology.
2. Only Dr. Hernández, the Project PI, and the Project Coordinator have master access to this server and the data.
3. Computer administrators, co-investigators, a project manager, project coordinators, and laboratory staff are only granted data access at the discretion of the Principal Investigator. Furthermore, this access is only granted as needed for finite periods of time throughout the study.
4. On a regular daily basis, laboratory staff destroy any “portable” data (including both electronic and paper copies) that are transferred from external data sources to the university computer server.
5. Once all records are linked and compiled into a single database, any and all PII identifiers will be destroyed.

The PI and the project coordinator are responsible for providing and documenting appropriate user access to the study database and preventing against major sources of data security problems: unauthorized internal access to data, external access to data, and malicious intent to destroy data and systems. This user access ensures that only appropriate and authorized personnel are able to view, access, and modify trial data.

Modifications to data will be performed in a manner that documents data modification, user access associated with modification, data associated with modification, and values prior to modification. The PI and the project coordinator will also be responsible for optimizing database performance, reliability, and backup of data. External, unauthorized access to data is prevented through cooperative efforts of the PI, the project manager, and network and systems administrators. Highly successful measures are employed through network firewall technologies to prevent unauthorized external access to data repositories.

### Confidentiality {27}

We establish excellent rapport with respondents and assure them that we are researchers with a private, local university and in no way representing NYCHA, law enforcement, or government agencies. No analyses, reports, or peer-reviewed articles will identify any participants. In-depth interviews may also be conducted somewhere nearby but not in the respondent’s household, such as a porch, church, park, and café, yet regardless of location all protections and confidentiality protocols apply.

We record information about the participant during initial participant recruitment allow us to recontact that same individual for follow-up surveys for the interim and long term assessments. With the participant’s consent, this information may include address and/or telephone number so that we can recontact them to schedule subsequent follow-up, possibly over the telephone. Participants are also informed that any other identifying information that is recorded—such as names and birthdates—for study participants, will be destroyed at the conclusion of the study.

### Plans for collection, laboratory evaluation, and storage of biological specimens for genetic or molecular analysis in this trial/future use {33}

Exposure to smoke either directly among smokers or indirectly through secondhand smoke will be assessed through self-report and salivary collection.

We collect saliva for 25% of recruited individuals from each building on a rolling basis at all assessment timepoints via passive drool collection. Donors tilt their head forward, allowing the saliva to pool on the floor of the mouth, then pass the saliva through the SalivaBio Collection Aid (SCA) into a polypropylene vial. Collection protocols/methods are available online at www.salimetrics.com or upon request (Salimetrics Cotinine ELISA Kits). Saliva cotinine will be measured at the Columbia University Biomarker Core Laboratory directed by Dr. Regina Santella using cotinine ELISA Kits (Salimetrics, State College, PA). The Salimetrics ELISA kit is an enzyme immunoassay used to measure primary or secondhand exposure to nicotine via cotinine. It is not intended for diagnostic use. It is intended only for research use among humans and some animals. The assay is non-invasive, involves no genetic sequencing, and is not used as a diagnostic procedure.

## Statistical methods

### Statistical methods for primary and secondary outcomes {20a}

We will conduct intent-to-treat (ITT) and contamination-adjusted intent-to-treat (CA-ITT) analyses to estimate the effects of the smoking relocation/cessation and resident endorsement interventions compared to the standard program implemented by NYCHA (no treatment). CA-ITT analyses will account for unanticipated contamination between trial arms and be completed via two-stage instrumental variables regressions using the original random allocation codes per assigned unit. Preliminary data analysis will include examination of distribution and summary statistics of all variables by intervention groups at each time point. The continuous variables with skewed distribution will be properly transformed, if necessary, to reduce impact of extreme values or improve model fitting. We will use box-plots to examine how distribution of a quantitative variable varies by categories of a categorical variable, and use scatter plots and Spearman correlation coefficient to examine bivariate associations between quantitative variables. Chi-square and Kruskal-Wallis tests will be used to detect group differences in categorical and quantitative baseline variables, respectively. For successful randomization, we expect no difference in the distribution of all baseline variables among the four arms. The baseline variables that differ by intervention groups and are related to outcomes of interest will be controlled in the aim-specific models.

#### Aim specific

We will use generalized linear models with repeated measures (GLMRM) for each aim where outcome variables may have different form (continuous, binary or count) and the measurements are likely to be correlated due to residing in the same building or being from the same participant over time. The models use various link functions to relate outcome to linear combination of predictors. For example, identity link for continuous outcome is linear model with repeated measures and logit link for binary outcome is logistic model with repeated measures. We will use the generalized estimation equation method to estimate model parameters and make statistical inference since it takes into account intra-cluster (within-building or within-person) correlations and uses all available data.

##### (1) Primary outcome

Self-reported average number of cigarettes smoked per day and secondhand smoking exposure (hours of secondhand smoke exposure in the building in the past 7 days) will be measured at baseline (in-person interview), 3 months (phone interview), and 12 months (in-person interview). Salivary cotinine will be measured for 25% of the sample at at least two timepoints across all assessment timepoints for smokers to evaluate reliability of self-reported smoking behavior in Aim 1 and for non-smokers to evaluate self-reported secondhand smoke exposure in Aim 2.

To examine the intervention group differences in the change of self-reported smoking (Aim 1), we will use GLMRM with identity link, *E*(*Yijkh*) = *ßh* + *ßkhtk* + *ßZij*, where *Yijkh* is an outcome variable (transformed if necessary) for smoking exposure measured at time *tk* (*t*0 =0 for baseline; *t*1 =1 for 6 months, 0 else; and *t*2 =1 for 1 year, 0 else) from the *i*th participant of the *j*th building in the *h*th arm/group (*h*=1, 2, 3, 4). For the reference group of *h*=4, *ßk*4 =0 for *k*=0, 1, 2; so that parameter *ßkh* (*k*=1, 2; *h*=1, 2, 3 for intervention arm) indicates the difference in outcome change since baseline between intervention group *h* and reference group, and *ß* is a vector of coefficients for the vector of control variables *Zij* (if any). To examine if effect of treatment A depends on treatment B or vice versa, we will test null hypothesis *ßk*1 = *ßk*2 + *ßk*3 for all *k*, which suggests independent effects of treatment A and treatment B and simpler model that *E*(*Yijk*) = *ß*0 + *ß*1A + *ß*2B + *ßk*0*tk* + *ßk*1*tk* A + *ßk*2*tk* B + *ßZij* with dummy variables A and B indicating treatment type. To examine the intervention group differences in the change in hours of secondhand smoke exposure in the building in the past 7 days for non-smokers, we will use GLMRM with log link (Aim 2). Models will be run with and without control variables (if any).

##### (2) Secondary outcome and sensitivity analyses

We will use GLMRM with logit or log links for binary or count outcomes, respectively, to evaluate group differences in the outcome changes from baseline to 3- and 12-month follow-up. The models will include predictors of dummy variables for time and group assignment as well as for group by time interaction as described above. Aim 1 (smoking behavior) secondary outcomes are whether smokers have successfully quit (binary), the mean number of quitting attempts (continuous). Aim 2 (exposure to secondhand smoke) secondary outcomes include ever observing someone smoking indoors within the building in the past 7 days (both smoker and non-smoker participants) (binary), hours of secondhand smoke exposure in the building in the past 7 days (count), number of people smoking, and number of cigarette butts in common areas of the building (count).

With regard to differential effect of the tobacco retail environment, the impact of differential tobacco retail environments (e.g., retailer density) around each building will be systematically evaluated for the primary outcomes and for quantitative secondary outcomes listed in Aims 1 and 2. The tobacco retail environment will be measured at the longitude-latitude point coordinate of each building using a geographic kernel density estimate with standard bandwidth to estimate the square mileage density of tobacco sellers in the surrounding area for each specific building point coordinate. This information is publicly available from the City of New York. As continuous metrics, these kernel density estimates will be separated in tertiles and at median breaks and the relationships tested in Aims 1 and 2 will then be tested within separate levels of tobacco retail density. We will also extend the GLMRM with identity or log link for each outcome variable in Aims 1 and 2 by adding the factor for retail environment, its interaction with group, time, and group by time interaction. In addition to the analysis for effect of a pre-specified modifier (the retail environment), we will conduct several a priori subgroup analyses to explore potential differential effect of the interventions in the continuous outcomes by age, sex, knowledge, and attitude towards the policy.

We will evaluate reliability of self-reported measures of active and passive smoking and revisit the primary analyses adjusting for potential imperfect reliability using regression calibration approaches.

### Interim analyses {21b}

Interim analyses are conducted periodically for quality assurance purposes. These interim analyses are reviewed by the research team to verify data quality and assess preliminary results.

### Methods for additional analyses (e.g., subgroup analyses) {20b}

Key informant (KI) interviews with individuals involved in building management and maintenance staff and those with expertise in the field (*n*= 20) were conducted to understand the current state of smoking at NYCHA sites and elicit their ideas about why smoking may persist in a mandated smoke-free context. We probed for recommended changes in the physical and social environments of the building including designated smoking areas and the information and support needed to more effectively support the implementation of the smoke-free policy.

Focus groups with 6–8 residents will be recruited via door knocking and lobby intercepts in non-study sites (*n*=3–8 groups) on an ongoing basis. Focus group participants (*n*= 24–64) are separated into English- and Spanish-led groups and discussion is conducted using a semi-structured interview guide to elicit their knowledge and perspectives of smoking and health in buildings and to provide recommendations and actionable steps to achieve greater compliance among fellow residents. Selecting non-study sites reduces contamination risk and engages more residents in discussions about smoke-free housing.

### Methods in analysis to handle protocol non-adherence and any statistical methods to handle missing data {20c}

Missing data, due to nonresponse or incomplete records, may lead to information biases. To account for the potential effects of missingness and data missing at random or with no known pattern, we will use multiple imputation techniques. Multiple imputation datasets of any missing data will be made under a joint model for the variable in question and a missingness indicator conditional on the fully observed data. Estimates obtained from the multiply imputed datasets will be combined using Rubin’s Rule (Little, R. J., & Rubin, D. B. (2019). Statistical analysis with missing data (Vol. 793). John Wiley & Sons.)

### Plans to give access to the full protocol, participant-level data, and statistical code {31c}

Data from approximately 512 participants will be released to sponsor (NIH) once the final dataset is analyzed and main findings are accepted for publication to support and validate study findings. This will include participant demographics, data from interviews, and laboratory data from saliva samples. Identifiers might be removed from the identifiable private information or identifiable biospecimens and that, after such removal, the information or biospecimens could be used for future research studies without additional consent from the subject. De-identified participant data may be utilized for the purposes of repeated analyses by other researchers to verify findings, and/or to promote further research with new or alternative hypotheses. The mechanism of distribution will be a data sharing agreement.

Access will be granted for researchers, institutions, and/or the broader public for as long as the data is anticipated to be useful. With the data sharing agreement in mind, the data can be made available for the duration of time needed to conduct analyses.

Access will be granted to those with a reputable background in the scientific field who have either a scientific or medical degree and/or a relevant position to ask for the data. Individuals should also express their intended use of the data. These requests will be routinely reviewed by the principal investigator [and/or designated team member].

## Oversight and monitoring

### Composition of the coordinating center and trial steering committee {5d}

**Fig. 6:**
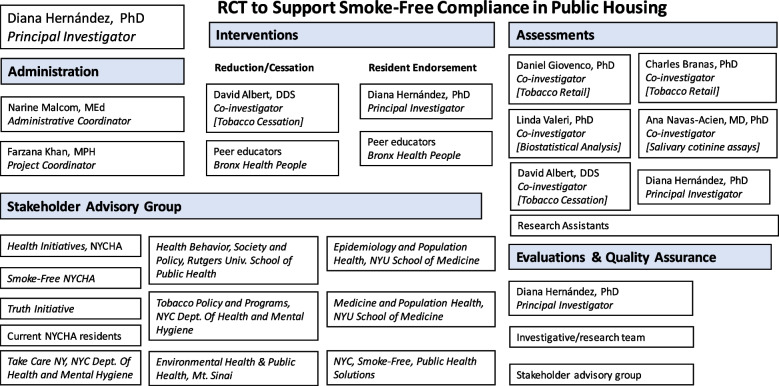
Composition of research team and stakeholders

The composition of the coordinating team and relevant partners are depicted in Fig. 6.

### Composition of the data monitoring committee, its role and reporting structure {21a}

All data monitoring for this study is overseen by the project principal investigator (Hernández) and is independent from the study sponsor, nor are there any competing interests; however, there is no formal data committee. Instead, process evaluations will be conducted via regular research team meetings and a stakeholder advisory board consisting of non-research personnel.

### Adverse event reporting and harms {22}

All adverse events will be reported to the principal investigator and subsequently to the IRB overseeing the study.

### Frequency and plans for auditing trial conduct {23}

Process evaluations includes checklists for intervention protocols and data collection packets, weekly meetings with data collectors, monthly investigator meetings, and quarterly advisory group communications via formal meetings and/or online engagements such as 1-1 meetings and newsletter distributions. These activities serve to periodically determine how well our study is operating. They also help to identify and resolve any problems or program needs. We survey participating residents and document the number of door magnets that remain on display over time, while also tracking activities on virtual platforms. These activities serve to document the organizational and operational procedures. A monthly quality assurance check and report is conducted by study staff to thoroughly review the following: recruitment, retention (follow-up), survey administration, quality of data collected, resource requirements and availability, barriers and facilitators to program implementation. We note any deviations from the original design and changes in roles/responsibilities of partner and collaborators. The results improve our program’s sustainability, help identify lessons learned and best practices, and result in key replication recommendations.

### Plans for communicating important protocol amendments to relevant parties (e.g., trial participants, ethical committees) {25}

All protocol modifications is reported to the overseeing IRB, investigating team, additional research personnel, participants, and registries in a timely fashion.

### Dissemination plans {31a}

The study has a strong focus on dissemination both during and after completion of the study. Throughout the duration of the study, investigators and additional research personnel consistently collaborate with NYCHA and engage with members of a stakeholder advisory board. Board members include residents, resident leaders, NYCHA personnel, community-based organization leaders among other key stakeholders in the smoke-free realm. These engagements are part of our process evaluation efforts to ensure appropriate study implementation.

Additionally, post data collection and data analyses, manuscripts will be written for publication with study results.

## Discussion

The research team have carefully considered several potential limitations of the RCT. We have implemented several design features to maximize recall including the use of structured questionnaires.

### Loss to follow-up and attrition bias

We will make every attempt to retain participants and minimize attrition bias through a range of rapport building and retention techniques, incentives, and cultural adaptation of study materials that we have successfully applied to past research [68–71]. We have also powered the study based on 80% retention.

### Information bias

Missing data, due to nonresponse or incomplete records, may lead to information biases. To account for the potential effects of missingness and data missing at random or with no known pattern, we will use multiple imputation techniques.

### Interviewer bias

We minimize interviewer biases, including social desirability bias, through highly structured questionnaires, training in standard probes, and other techniques. To specifically minimize social desirability bias, we use introductory language to reinforce the neutrality of our interviewers and the questions they are administering as well as standardized questions to measure social desirability [72]. We also do not inform the interviewers of the study hypotheses and employ blinding wherever possible.

### Treatment spillover or diffusion of treatment onto control group sites

We recognize that treatment spillover or diffusion of treatment onto control group sites may be a threat to internal validity [73] and as such, all buildings assigned to an intervention will be in separate developments from the control group sites. We will also conduct specific statistical analyses of contamination-adjusted ITT effects [74, 75] and spillover effects as done successfully in numerous prior studies [73, 76, 77].

We also acknowledge the limitations in the implementation of the reduction/cessation intervention. Utilizing peer educators limits the degree of customization and breadth of services and support provided to participants in the R/C and R/C+RE arms. Though some customization is possible depending on participant needs, the curriculum is standardized and simplified. Though the PEs do not have clinical expertise or training and, thus, are limited in the degree of support they can provide, efforts to incorporate peers may empower residents and further advance community engagement efforts.

### Strengths

This RCT leverages a monumental policy shift that affects nearly half a million New York City (NYC) public housing residents, many of whom disproportionately experience chronic illness and are more likely to smoke and be exposed to secondhand smoke than other city residents. This first-ever RCT tests the effects of much-needed compliance strategies on resident smoking behavior and secondhand smoke exposure in multiunit housing. We are testing interventions at the building level that are framed by a broad, system-wide and national-level smoking ban in public housing, Medicaid-supported smoking cessation, and variations in the tobacco retail environment in neighborhoods throughout NYC. Within the scope of a changing policy landscape and differing community contexts, a series of public housing building clusters will receive different policy compliance interventions, focusing on either reduction/cessation or the resident endorsement of smoke-free homes that will be tested in terms of longitudinal pre-post outcomes among residents who smoke and those that do not smoke. These interventions seek to enhance policy compliance, have been previously piloted, are adaptable to public housing settings around the country given the national ban, and address tobacco disparities while producing lasting health benefits at relatively low costs.

Further, this study has implications for the allocation of resources related to the smoke-free policy extending beyond NYCHA to other public housing located nationwide by providing evidence-based support for efficacious methods to reduce personal smoking and exposure to secondhand smoke. Our findings have the potential to affect comprehensive and interlinked tobacco control policies at federal, state, and local levels (i.e., nationwide smoke-free housing policy implementation in public housing, NY State Medicaid coverage for smoking cessation, and local legislation limiting licensing that will affect the tobacco retail environment). As such, the study has potential to drive better health outcomes overall for public housing residents.

## Trial status

Protocol Version September 2022

Recruitment start date: February 3, 2022

Approximate date of recruitment completion: August 2023

## Data Availability

Data from the research project will be released to our sponsor (NIH) once the final dataset is analyzed and main findings are accepted for publication. This data will include data from participants’ interviews and laboratory results. We will remove identifiable information from participants’ private information and/or their identifiable biospecimens. Once this is completed, the de-identified data and/or samples may be utilized for repeated analyses by other researchers to verify findings, and/or to promote further research with new or alternative hypotheses. Upon request, we will use a data sharing agreement prior to providing the information to researchers, institutions, and/or the broader public. This will be done and reviewed by the principal investigator to establish the intended use of the data and screen the requests to ensure the data is shared with appropriate individuals.

## Abbreviations

HUD: Housing and Urban Development
RCT: Randomized control trial
NYCHA: New York City Housing Authority
CBO: Community-based organization
RAD: Rental Assistance Demonstration
PACT: Permanent Affordability Commitment Together
PE: Peer educator
R/C: Reduction (via relocation and reduction in personal smoking)/cessation
RE: Resident endorsement
NYC: New York City
